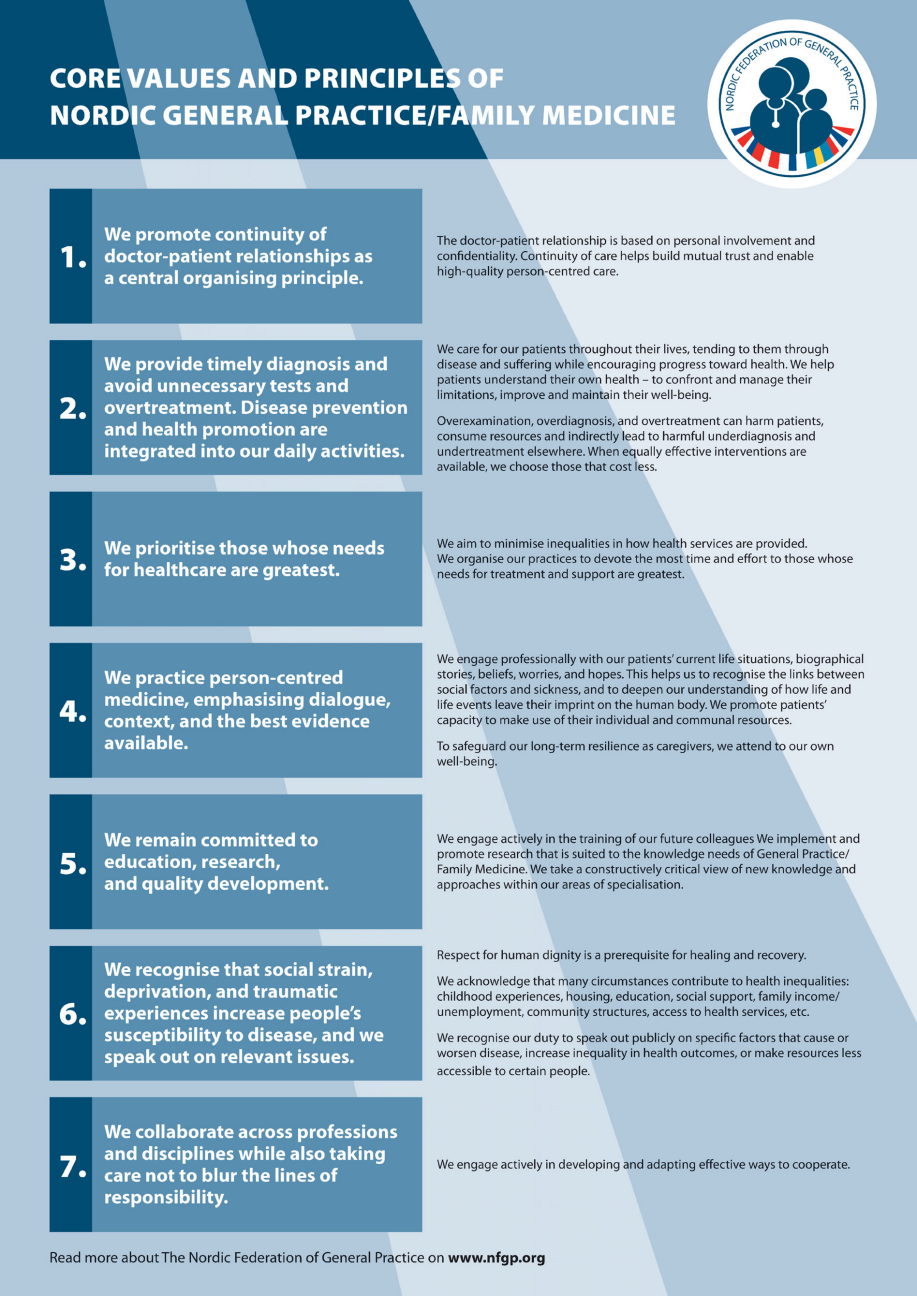# Core Values and Principles of Nordic General Practice/Family Medicine

**DOI:** 10.1080/02813432.2020.1842674

**Published:** 2020-12-07

**Authors:** Nordic Federation of General Practice (NFGP)

WHO (https://www.who.int/docs/default-source/primary-health/declaration/gcphc-declaration.pdf) considers primary health care to be a cornerstone of sustainable health care systems. General Practice/Family Medicine is the key provider of primary health care.

WONCA Europe (https://www.woncaeurope.org/file/520e8ed3-30b4-4a74-bc35-87286d3de5c7/Definition 3rd ed 2011 with revised wonca tree.pdf) has defined General Practice/Family Medicine as both a clinical specialty and a discipline in its own right, with its own distinct curriculum and research base.

As an academic discipline, General Practice/Family Medicine is based on knowledge and methodology drawn from the Natural sciences as well as the Humanities. ***As committed leaders*** *in the ongoing process of defining and implementing core values and principles, general practitioners aim to:*

- promote and protect the health and well-being of each individual patient while keeping in mind the needs of the general population;
- provide a frame of reference for our professional identity;
- provide a basis for continuing professional development, with curricula and training adapted to every educational level – undergraduate, post-graduate, and beyond;
- communicate our mandate and the principles of our work to patients, fellow healthcare workers, and the communities we serve.

**Figure:**